# [*N*,*N*′-Bis(4-chloro­phen­yl)pentane-2,4-diiminato]dicarbonyl­rhodium(I)

**DOI:** 10.1107/S1600536810000449

**Published:** 2010-01-27

**Authors:** T. N. Hill, G. Steyl

**Affiliations:** a Department of Chemistry, University of the Free State, Bloemfontein 9300, South Africa

## Abstract

The title compound, [Rh(C_17_H_15_Cl_2_N_2_)(CO)_2_], is a rhodium(I) derivative of a β-diketiminato moiety. It is an example of a new type of β-diketiminate derivative that has not yet been characterized *via* solid-state methods. The complex crystallizes with a distorted square-planar geometry about the Rh^I^ atom (*m* symmetry). A weak inter­molecular C—H⋯O contact is observed.

## Related literature

For related diketiminato complexes, see: Smith *et al.* (2002 ▶, 2006 ▶).
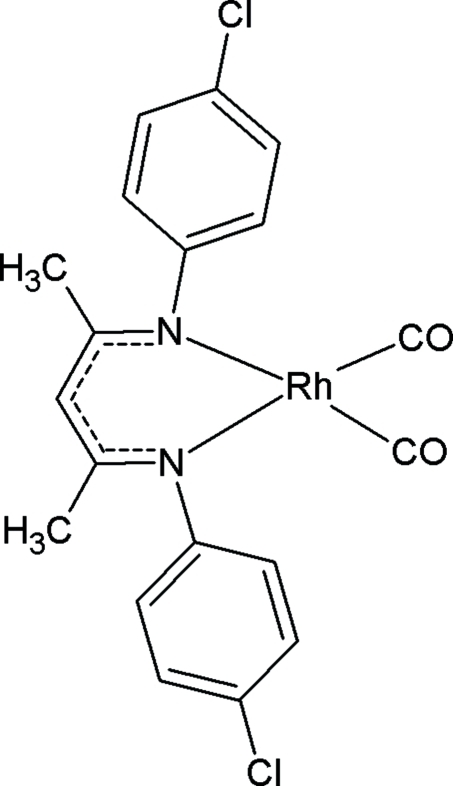


## Experimental

### 

#### Crystal data

[Rh(C_17_H_15_Cl_2_N_2_)(CO)_2_]
*M* *_r_* = 477.14
Monoclinic, 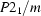

*a* = 9.6726 (3) Å
*b* = 7.5911 (2) Å
*c* = 13.6484 (4) Å
β = 107.247 (1)°
*V* = 957.08 (5) Å^3^
*Z* = 2
Mo *K*α radiation
μ = 1.19 mm^−1^
*T* = 100 K
0.36 × 0.31 × 0.30 mm

#### Data collection

Bruker X8 APEXII 4K Kappa CCD diffractometer
Absorption correction: multi-scan (*SADABS*; Bruker, 2004 ▶) *T*_min_ = 0.675, *T*_max_ = 0.717
27556 measured reflections
2555 independent reflections
2491 reflections with *I* > 2σ(*I*)
*R*_int_ = 0.026

#### Refinement

*R*[*F* ^2^ > 2σ(*F* ^2^)] = 0.017
*wR*(*F* ^2^) = 0.050
*S* = 0.93
2555 reflections
149 parameters
H atoms treated by a mixture of independent and constrained refinement
Δρ_max_ = 0.36 e Å^−3^
Δρ_min_ = −0.85 e Å^−3^

### 

Data collection: *APEX2* (Bruker, 2005 ▶); cell refinement: *SAINT-Plus* (Bruker, 2004 ▶); data reduction: *SAINT-Plus* and *XPREP* (Bruker, 2004 ▶); program(s) used to solve structure: *SHELXS97* (Sheldrick, 2008 ▶); program(s) used to refine structure: *SHELXL97* (Sheldrick, 2008 ▶); molecular graphics: *DIAMOND* (Brandenburg & Putz, 2006 ▶); software used to prepare material for publication: *WinGX* (Farrugia, 1999 ▶).

## Supplementary Material

Crystal structure: contains datablocks global, I. DOI: 10.1107/S1600536810000449/gw2073sup1.cif

Structure factors: contains datablocks I. DOI: 10.1107/S1600536810000449/gw2073Isup2.hkl

Additional supplementary materials:  crystallographic information; 3D view; checkCIF report

## Figures and Tables

**Table:** 

Rh1—C02	1.854 (2)
Rh1—C01	1.8741 (19)
Rh1—N1	2.0453 (14)
Rh1—N2	2.0523 (14)
O01—C01	1.134 (2)
O02—C02	1.137 (3)

**Table:** 

C02—Rh1—C01	85.48 (8)
C02—Rh1—N1	91.89 (7)
C01—Rh1—N2	92.76 (7)
N1—Rh1—N2	89.87 (6)
O01—C01—Rh1	176.21 (17)
O02—C02—Rh1	177.45 (18)

**Table 2:** Hydrogen-bond geometry (Å, °)

*D*—H⋯*A*	*D*—H	H⋯*A*	*D*⋯*A*	*D*—H⋯*A*
C22—H22⋯O02^i^	0.95	2.54	3.1723 (18)	124

Symmetry code: (i) 
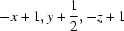
.

**Fig. 1.**
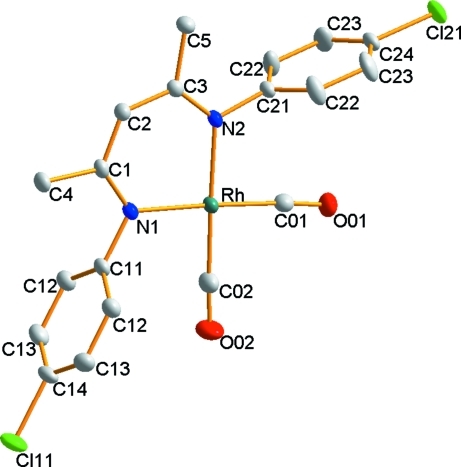
View of (I) (50% probability displacement ellipsoids)

## supplementary crystallographic information

### Comment

In literature similar iron complexes have been reported containing tertiary butyl, isopropyl and methyl derivatives (Smith *et al.*, 2002; Smith *et al.*, 2006). Using a comparable ligand system, with electron withdrawing substituents on the phenyl ring, a series of rhodium(I) dicarbonyl complexes were prepared. The title compound (I) is a novel example of a 4-Chlorophenyl derivative. (Figure 1)

Due to the highly symmetrical nature of the complex both the Rh—C and Rh—N bond distances are similar. The carbonyl oxygen bond distances are the same, with the carbonyl's themselves being close to linearity. The mirror plane bisects the complex passing through the metal centre, the ketimine backbone and carbonyl moeities. (Table 1)

A staggared head-to-tail stacking is observed with no Rh—Rh interaction. The closest contact is a weak hydrogen bond between the phenyl ring (C22) of the diketiminato ligand and the adjacent carbonyl oxygen O02 (Table 2).

### Experimental

The title complex was synthesized by the addition of *N*,*N*'-bis-(4-chlorophenyl)pentane-2,4-di-imine (167 mg, 0.514 mmol) to an acetone solution of the [Rh(µ-Cl)(CO)_2_]_2_ (100 mg, 0.257 mmol). On slow evaporation of the solvent; crystals suitable for X-Ray crystallography were obtained. Yield: 147 mg (60%).

### Refinement

All H atoms were positioned geometrically and allowed to ride on their parent atoms, with *U*_iso_(H) = 1.2U_eq(parent)_ of the parent atom with a C—H distance of 0.99 (methyl) and 0.95 (aromatic).

### Crystal data

**Table:**

[Rh(C_17_H_15_Cl_2_N_2_)(CO)_2_]	*F*(000) = 476
*M**_r_* = 477.14	*D*_x_ = 1.656 Mg m^−^^3^
Monoclinic, *P*2_1_/*m*	Mo *K*α radiation, λ = 0.71073 Å
Hall symbol: -P 2yb	Cell parameters from 7852 reflections
*a* = 9.6726 (3) Å	θ = 2.7–28.3°
*b* = 7.5911 (2) Å	µ = 1.19 mm^−^^1^
*c* = 13.6484 (4) Å	*T* = 100 K
β = 107.247 (1)°	Cuboid, colourless
*V* = 957.08 (5) Å^3^	0.36 × 0.31 × 0.3 mm
*Z* = 2	

### Data collection

**Table:**

Bruker X8 APEXII 4K Kappa CCD diffractometer	2555 independent reflections
Radiation source: sealed tube	2491 reflections with *I* > 2σ(*I*)
graphite	*R*_int_ = 0.026
Detector resolution: 512 pixels mm^-1^	θ_max_ = 28.3°, θ_min_ = 2.2°
ω and φ scans	*h* = −12→12
Absorption correction: multi-scan (*SADABS*; Bruker, 2004)	*k* = −10→10
*T*_min_ = 0.675, *T*_max_ = 0.717	*l* = −18→15
27556 measured reflections	

### Refinement

**Table:**

Refinement on *F*^2^	0 restraints
Least-squares matrix: full	H atoms treated by a mixture of independent and constrained refinement
*R*[*F*^2^ > 2σ(*F*^2^)] = 0.017	*w* = 1/[σ^2^(*F*_o_^2^) + (0.0354*P*)^2^ + 0.5478*P*] where *P* = (*F*_o_^2^ + 2*F*_c_^2^)/3
*wR*(*F*^2^) = 0.050	(Δ/σ)_max_ = 0.001
*S* = 0.93	Δρ_max_ = 0.36 e Å^−^^3^
2555 reflections	Δρ_min_ = −0.85 e Å^−^^3^
149 parameters	

### Special details

**Table:**

Geometry. All e.s.d.'s (except the e.s.d. in the dihedral angle between two l.s. planes) are estimated using the full covariance matrix. The cell e.s.d.'s are taken into account individually in the estimation of e.s.d.'s in distances, angles and torsion angles; correlations between e.s.d.'s in cell parameters are only used when they are defined by crystal symmetry. An approximate (isotropic) treatment of cell e.s.d.'s is used for estimating e.s.d.'s involving l.s. planes.

### Fractional atomic coordinates and isotropic or equivalent isotropic displacement parameters (Å^2^)

**Table:**

	*x*	*y*	*z*	*U*_iso_*/*U*_eq_	Occ. (<1)
Rh1	0.637102 (13)	0.25	0.465229 (9)	0.01730 (6)	
N1	0.70723 (16)	0.25	0.33783 (11)	0.0184 (3)	
N2	0.84799 (16)	0.25	0.55685 (11)	0.0174 (3)	
O01	0.51242 (16)	0.25	0.64255 (11)	0.0306 (3)	
O02	0.32539 (17)	0.25	0.34080 (13)	0.0470 (5)	
Cl11	0.25234 (6)	0.25	−0.06636 (4)	0.04074 (14)	
Cl21	0.87899 (6)	0.25	0.99767 (3)	0.03319 (12)	
C01	0.5641 (2)	0.25	0.57800 (15)	0.0233 (4)	
C1	0.84413 (19)	0.25	0.33584 (13)	0.0183 (3)	
C02	0.4448 (2)	0.25	0.38608 (15)	0.0298 (4)	
C2	0.96380 (18)	0.25	0.42351 (13)	0.0185 (3)	
H2	1.0557	0.25	0.4116	0.022*	
C3	0.96652 (19)	0.25	0.52655 (13)	0.0178 (3)	
C4	0.8736 (2)	0.25	0.23319 (13)	0.0231 (4)	
H4A	0.820 (4)	0.152 (3)	0.1908 (14)	0.035*	0.5
H4B	0.9787 (16)	0.235 (5)	0.2437 (2)	0.035*	0.5
H4C	0.841 (4)	0.363 (3)	0.1977 (13)	0.035*	0.5
C5	1.11412 (19)	0.25	0.60505 (14)	0.0221 (3)	
H5A	1.1388 (9)	0.364 (2)	0.6276 (10)	0.033*	0.5
H5B	1.1816 (12)	0.206 (2)	0.5756 (6)	0.033*	0.5
H5C	1.1122 (5)	0.180 (2)	0.6599 (12)	0.033*	0.5
C11	0.59706 (19)	0.25	0.23963 (13)	0.0206 (3)	
C12	0.54311 (14)	0.09167 (18)	0.19293 (10)	0.0243 (3)	
H12	0.5792	−0.0165	0.2255	0.029*	
C13	0.43608 (14)	0.0908 (2)	0.09835 (10)	0.0278 (3)	
H13	0.3988	−0.0172	0.066	0.033*	
C14	0.3853 (2)	0.25	0.05267 (15)	0.0281 (4)	
C21	0.86559 (18)	0.25	0.66523 (12)	0.0185 (3)	
C22	0.86893 (17)	0.40678 (19)	0.71687 (10)	0.0303 (3)	
H22	0.8683	0.5151	0.6819	0.036*	
C23	0.87317 (17)	0.4078 (2)	0.81978 (10)	0.0322 (3)	
H23	0.8749	0.516	0.8552	0.039*	
C24	0.8748 (2)	0.25	0.86918 (13)	0.0232 (4)	

### Atomic displacement parameters (Å^2^)

**Table:**

	*U*^11^	*U*^22^	*U*^33^	*U*^12^	*U*^13^	*U*^23^
Rh1	0.01599 (8)	0.02468 (8)	0.01164 (8)	0	0.00473 (5)	0
N1	0.0194 (7)	0.0248 (7)	0.0112 (6)	0	0.0046 (5)	0
N2	0.0191 (7)	0.0228 (7)	0.0102 (6)	0	0.0044 (5)	0
O01	0.0300 (7)	0.0437 (8)	0.0230 (7)	0	0.0154 (6)	0
O02	0.0194 (7)	0.0851 (14)	0.0326 (9)	0	0.0018 (6)	0
Cl11	0.0270 (2)	0.0652 (4)	0.0215 (2)	0	−0.00592 (18)	0
Cl21	0.0342 (2)	0.0550 (3)	0.01086 (19)	0	0.00754 (17)	0
C01	0.0192 (8)	0.0295 (9)	0.0207 (8)	0	0.0052 (7)	0
C1	0.0230 (8)	0.0206 (7)	0.0123 (7)	0	0.0068 (6)	0
C02	0.0240 (9)	0.0461 (12)	0.0206 (9)	0	0.0087 (7)	0
C2	0.0175 (7)	0.0238 (8)	0.0151 (7)	0	0.0063 (6)	0
C3	0.0194 (8)	0.0187 (7)	0.0150 (7)	0	0.0047 (6)	0
C4	0.0241 (9)	0.0332 (9)	0.0138 (8)	0	0.0081 (7)	0
C5	0.0184 (8)	0.0298 (9)	0.0170 (8)	0	0.0038 (6)	0
C11	0.0201 (8)	0.0305 (9)	0.0116 (7)	0	0.0055 (6)	0
C12	0.0253 (6)	0.0302 (6)	0.0170 (5)	−0.0013 (5)	0.0056 (5)	−0.0004 (5)
C13	0.0255 (6)	0.0376 (7)	0.0196 (6)	−0.0056 (6)	0.0055 (5)	−0.0053 (5)
C14	0.0186 (8)	0.0500 (13)	0.0136 (7)	0	0.0013 (6)	0
C21	0.0171 (7)	0.0268 (8)	0.0116 (7)	0	0.0044 (6)	0
C22	0.0500 (8)	0.0255 (6)	0.0179 (6)	−0.0132 (6)	0.0140 (6)	−0.0030 (5)
C23	0.0489 (8)	0.0319 (7)	0.0182 (6)	−0.0150 (6)	0.0135 (6)	−0.0094 (5)
C24	0.0203 (8)	0.0391 (10)	0.0098 (7)	0	0.0041 (6)	0

### Geometric parameters (Å, °)

**Table:**

Rh1—C02	1.854 (2)	C4—H4C	0.9902
Rh1—C01	1.8741 (19)	C5—H5A	0.9234
Rh1—N1	2.0453 (14)	C5—H5B	0.9234
Rh1—N2	2.0523 (14)	C5—H5C	0.9234
N1—C1	1.332 (2)	C11—C12	1.3877 (16)
N1—C11	1.444 (2)	C11—C12^i^	1.3877 (16)
N2—C3	1.329 (2)	C12—C13	1.3949 (17)
N2—C21	1.438 (2)	C12—H12	0.95
O01—C01	1.134 (2)	C13—C14	1.3816 (18)
O02—C02	1.137 (3)	C13—H13	0.95
Cl11—C14	1.748 (2)	C14—C13^i^	1.3816 (18)
Cl21—C24	1.7420 (18)	C21—C22	1.3787 (16)
C1—C2	1.397 (2)	C21—C22^i^	1.3787 (16)
C1—C4	1.510 (2)	C22—C23	1.3929 (17)
C2—C3	1.399 (2)	C22—H22	0.95
C2—H2	0.95	C23—C24	1.3727 (17)
C3—C5	1.510 (2)	C23—H23	0.95
C4—H4A	0.9902	C24—C23^i^	1.3727 (17)
C4—H4B	0.9902		
			
C02—Rh1—C01	85.48 (8)	C3—C5—H5B	109.5
C02—Rh1—N1	91.89 (7)	H5A—C5—H5B	109.5
C01—Rh1—N1	177.37 (6)	C3—C5—H5C	109.5
C02—Rh1—N2	178.24 (7)	H5A—C5—H5C	109.5
C01—Rh1—N2	92.76 (7)	H5B—C5—H5C	109.5
N1—Rh1—N2	89.87 (6)	C12—C11—C12^i^	120.01 (16)
C1—N1—C11	116.46 (14)	C12—C11—N1	119.99 (8)
C1—N1—Rh1	126.83 (12)	C12^i^—C11—N1	119.99 (8)
C11—N1—Rh1	116.72 (11)	C11—C12—C13	120.27 (13)
C3—N2—C21	118.04 (14)	C11—C12—H12	119.9
C3—N2—Rh1	127.13 (12)	C13—C12—H12	119.9
C21—N2—Rh1	114.83 (11)	C14—C13—C12	118.70 (14)
O01—C01—Rh1	176.21 (17)	C14—C13—H13	120.7
N1—C1—C2	123.98 (15)	C12—C13—H13	120.7
N1—C1—C4	118.73 (15)	C13^i^—C14—C13	122.04 (18)
C2—C1—C4	117.29 (16)	C13^i^—C14—Cl11	118.98 (9)
O02—C02—Rh1	177.45 (18)	C13—C14—Cl11	118.98 (9)
C1—C2—C3	128.69 (16)	C22—C21—C22^i^	119.37 (16)
C1—C2—H2	115.7	C22—C21—N2	120.24 (8)
C3—C2—H2	115.7	C22^i^—C21—N2	120.24 (8)
N2—C3—C2	123.51 (15)	C21—C22—C23	120.65 (13)
N2—C3—C5	120.04 (15)	C21—C22—H22	119.7
C2—C3—C5	116.45 (15)	C23—C22—H22	119.7
C1—C4—H4A	109.5	C24—C23—C22	118.87 (14)
C1—C4—H4B	109.5	C24—C23—H23	120.6
H4A—C4—H4B	109.5	C22—C23—H23	120.6
C1—C4—H4C	109.5	C23^i^—C24—C23	121.59 (17)
H4A—C4—H4C	109.5	C23^i^—C24—Cl21	119.20 (8)
H4B—C4—H4C	109.5	C23—C24—Cl21	119.20 (8)
C3—C5—H5A	109.5		
			
C02—Rh1—N1—C1	180	C1—C2—C3—C5	180
N2—Rh1—N1—C1	0	C1—N1—C11—C12	90.64 (14)
C02—Rh1—N1—C11	0	Rh1—N1—C11—C12	−89.36 (14)
N2—Rh1—N1—C11	180	C1—N1—C11—C12^i^	−90.64 (14)
C01—Rh1—N2—C3	180	Rh1—N1—C11—C12^i^	89.36 (14)
N1—Rh1—N2—C3	0	C12^i^—C11—C12—C13	0.9 (3)
C01—Rh1—N2—C21	0	N1—C11—C12—C13	179.63 (13)
N1—Rh1—N2—C21	180	C11—C12—C13—C14	−0.1 (2)
C11—N1—C1—C2	180	C12—C13—C14—C13^i^	−0.8 (3)
Rh1—N1—C1—C2	0	C12—C13—C14—Cl11	179.46 (11)
C11—N1—C1—C4	0	C3—N2—C21—C22	92.31 (15)
Rh1—N1—C1—C4	180	Rh1—N2—C21—C22	−87.69 (15)
N1—C1—C2—C3	0	C3—N2—C21—C22^i^	−92.31 (15)
C4—C1—C2—C3	180	Rh1—N2—C21—C22^i^	87.69 (15)
C21—N2—C3—C2	180	C22^i^—C21—C22—C23	−1.1 (3)
Rh1—N2—C3—C2	0	N2—C21—C22—C23	174.34 (15)
C21—N2—C3—C5	0	C21—C22—C23—C24	0.4 (3)
Rh1—N2—C3—C5	180	C22—C23—C24—C23^i^	0.4 (3)
C1—C2—C3—N2	0	C22—C23—C24—Cl21	−179.62 (13)

Symmetry codes: (i) *x*, −*y*+1/2, *z*.

### Hydrogen-bond geometry (Å, °)

**Table:**

*D*—H···*A*	*D*—H	H···*A*	*D*···*A*	*D*—H···*A*
C22—H22···O02^ii^	0.95	2.54	3.1723 (18)	124

Symmetry codes: (ii) −*x*+1, *y*+1/2, −*z*+1.
